# Whole-genome sequencing identifies novel loci for keratoconus and facilitates risk stratification in a Han Chinese population

**DOI:** 10.1186/s40662-024-00421-1

**Published:** 2025-01-06

**Authors:** Yinghao Yao, Xingyong Li, Lan Wu, Jia Zhang, Yuanyuan Gui, Xiangyi Yu, Yang Zhou, Xuefei Li, Xinyu Liu, Shilai Xing, Gang An, Zhenlin Du, Hui Liu, Shasha Li, Xiaoguang Yu, Myopia Associated Genetics Intervention and Consortiums, Hua Chen, Jianzhong Su, Shihao Chen

**Affiliations:** 1 Oujiang Laboratory (Zhejiang Lab for Regenerative Medicine, Vision and Brain Health), Eye Hospital, Wenzhou Medical University, Wenzhou, 325000 Zhejiang China; 2 National Engineering Research Center of Ophthalmology and Optometry, Eye Hospital, Wenzhou Medical University, Wenzhou, 325027 China; 3 National Clinical Research Center for Ocular Diseases, Eye Hospital, Wenzhou Medical University, Wenzhou, 325027 China; 4 Beijing Institute of Genomics, Chinese Academy of Sciences and China National Center for Bioinformation, Beijing, 100101 China; 5 University of Chinese Academy of Sciences, Beijing, 100049 China; 6 Institute of PSI Genomics, Wenzhou Global Eye & Vision Innovation Center, Wenzhou, 325024 China; 7 Taizhou Eye Hospital, Taizhou, 318001 China

**Keywords:** Keratoconus, Han Chinese, Whole-genome sequencing, Genome wide association analysis, Polygenic risk prediction

## Abstract

**Background:**

Keratoconus (KC) is a prevalent corneal condition with a modest genetic basis. Recent studies have reported significant genetic associations in multi-ethnic cohorts. However, the situation in the Chinese population remains unknown. This study was conducted to identify novel genetic variants linked to KC and to evaluate the potential applicability of a polygenic risk model in the Han Chinese population.

**Methods:**

A total of 830 individuals diagnosed with KC and 779 controls from a Chinese cohort were enrolled and genotyped by whole-genome sequencing (WGS). Common and rare variants were respectively subjected to single variant association analysis and gene-based burden analysis. Polygenic risk score (PRS) models were developed using top single-nucleotide polymorphisms (SNPs) identified from a multi-ethnic meta-analysis and then evaluated in the Chinese cohort.

**Results:**

The characterization of germline variants entailed correction for population stratification and validation of the East Asian ancestry of the included samples via principal component analysis. For rare protein-truncating variants (PTVs) with minor allele frequency (MAF) < 5%, *ZC3H11B* emerged as the top prioritized gene, albeit failing to reach the significance threshold. We detected three common variants reaching genome-wide significance (*P* ≤ 5 × 10^−8^), all of which are novel to KC. Our study validated three well known predisposition loci, *COL5A1*, *EIF3A* and *FNDC3B*. Additionally, a significant correlation of allelic effects was observed for suggestive SNPs between the largest multi-ethnic meta-genome-wide association study (GWAS) and our study. The PRS model, generated using top SNPs from the meta-GWAS, stratified individuals in the upper quartile, revealing up to a 2.16-fold increased risk for KC.

**Conclusions:**

Our comprehensive WGS-based GWAS in a large Chinese cohort enhances the efficiency of array-based genetic studies, revealing novel genetic associations for KC and highlighting the potential for refining clinical decision-making and early prevention strategies.

**Supplementary Information:**

The online version contains supplementary material available at 10.1186/s40662-024-00421-1.

## Background

Keratoconus (KC) is almost always a bilateral progressive corneal disease, usually asymmetrical, and is influenced by genetic factors (such as family history) and environmental factors (like eye rubbing and nocturnal ocular compression). It is no longer regarded as a non-inflammatory condition, as numerous pro-inflammatory factors have been associated with its development [1–5]. The disease is characterized by thinning and steepening of the paracentral cornea, resulting in progressively irregular astigmatism, which eventually may lead to severe visual impairment and even legal blindness [6, 7]. The estimated prevalence of KC can vary depending on the population, typically ranging from 1.2% in some predominantly European populations [8] to 2.3%–3.3% in some East or South Asian populations [9, 10]. Notably, due mainly to self-selection bias, the proportion of refractive surgery candidates with KC or suspected KC has been found to be particularly high, reaching up to 32.3% in a study conducted in Saudi Arabia [11]. Therefore, early diagnosis of KC holds considerable clinical significance.

A high occurrence rate in first-degree relatives and concordance in twins indicate a substantial genetic component in KC [12, 13]. Linkage studies and genome-wide association studies (GWAS) have revealed multiple loci associated with central corneal thickness (CCT) that are also linked to an increased risk of KC [14–17]. Previous studies have also implicated single nucleotide polymorphism (SNP) alleles upstream of the *ZNF469* locus, which is associated with a higher CCT but an increased risk for KC [17–19]. These findings emphasize the distinct genetic foundations of CCT and KC, where CCT remains relatively stable over time, in contrast to the acquired and progressive corneal thinning characteristic of KC [20].

The largest multi-ethnic GWAS for KC conducted to date, involving 4669 cases and 116,547 controls, has revealed 36 significant risk regions. These regions include associations near or within genes that encode for fibrillar collagens (types I and V), microfibrillar (VI), and peri-fibrillar (XII) structures [21]. While earlier genetic studies of KC successfully identified several variants in known genes, including *VSX1* [22–24], *TGFB1* [25], *COL4A3* [25, 26], *DOCK9* [27], and *LOX* [28], they were able to explain only a small fraction of the phenotypic variants associated with the condition. Furthermore, it is noteworthy that the majority of previous studies have predominantly concentrated on the European population using SNP arrays, limiting generalizability of their findings and may not fully capture the diversity of genetic factors contributing to KC across different populations. Therefore, there is a need for more diverse and comprehensive investigations to gain a broader understanding of the genetic underpinnings of this complex condition.

Here, we conducted the first large-scale whole-genome sequencing (WGS) on 830 individuals diagnosed with KC and 779 control individuals of Han Chinese ancestry. For rare variants [minor allele frequency (MAF) < 5%], we performed collapsing-based burden tests at both the gene and gene-set levels. Single-variant association analyses of common and low-frequency variants (MAF > 5%) were executed after controlling for population sub-structure. Finally, we devised a cross-ancestry polygenic risk score (PRS) to assess its utility in risk stratification within the Chinese population.

## Methods

### Study subjects

This study enrolled 830 KC patients and 779 controls of Han Chinese ancestry from the Eye Hospital of Wenzhou Medical University, spanning the period from September 2014 and August 2023. All participants underwent a comprehensive ophthalmic assessment, including manifest refraction, slit-lamp biomicroscopy examination, and corneal tomography evaluation using Pentacam HR (Oculus GmbH, Wetzlar, Germany). The diagnosis of KC is established based on the following criteria: (1) presence of at least one typical clinical feature of KC, such as Fleischer’s ring, Vogt’s striae, anterior stromal scar, localized stromal thinning, or conical protrusion; (2) typical abnormal topographic findings, including asymmetric bow tie, posterior or anterior focal steepening; and (3) abnormal topographic indices, such as an inferior–superior index > 1.5, maximum keratometry (Kmax) > 47 D, and a difference in Kmax between the two eyes > 1 D [29]. The definition of the case group requires meeting all three criteria: conditions 1 and 2 must be fully satisfied, while at least one criterion from condition 3 is sufficient. Patients with secondary KC, a family history of KC, or related syndromes were excluded from the study. The control group was required to be free of a KC diagnosis.

This study was conducted according to the Declaration of Helsinki and approved by the Institutional Review Board of The Eye Hospital of Wenzhou Medical University (2023-071-K-59). Written informed consent was obtained from all subjects.

### Whole genome sequencing, variants calling and genotyping

The genomic DNA of all subjects was isolated from peripheral blood via standard procedures using the Magen IVD3018 kit. The WGS was performed using DNBSEQ-T7. Raw sequencing reads were assessed by the FastQC package and trimmed with TrimGalore (https://github.com/FelixKrueger/TrimGalore) to remove poor quality reads and adapter contamination. Clean reads were mapped to the human reference genome (GRCh38) using Burrows-Wheeler Aligner (BWA-MEM v0.7.15) [30] with default parameters. Unmapped reads were excluded based on the flag field using samtools [31], and reads with a mapping quality (MAPQ) below 20 were similarly filtered out. Variants were prefiltered so that only those passing the Genome Analysis Toolkit (GATK) variant quality score recalibration (VQSR) metric and those located outside of low-complexity regions remained [32].

### Genotype and variants quality control

A quality control process consisting multiple steps has been designed to ensure the reliability of subsequent association analysis results (Supplementary Fig. 1). First, variants were prefiltered if their average coverage < 8 and heterozygous genotype calls with an allele balance > 0.8 or < 0.2 were set as missing. We excluded variants with a call rate < 0.98, a case–control call rate difference > 0.005, and a Hardy–Weinberg equilibrium (HWE) test *P* < 10^−6^ on the controls and < 10^−10^ for cases. In the single-variant association analysis, only biallelic variants with a MAF > 0.05 were included due to our small sample size. Samples were excluded if they showed a low average call rate < 0.98 and low mean sequencing depth (DP) < 8. In addition, samples with heterozygosity F deviating more than 3 standard deviation (SD) and relationships between individuals with a pihat > 0.2 were further excluded.

For rare protein-coding variants, we applied stringent filtering criteria, including a requirement for genotype quality (GQ) ≥ 30, a minimum DP of ≥ 20, and a call rate of ≥ 90%. Additionally, variants were restricted to those with a MAF < 0.001 specifically within the East Asian population as reported in gnomAD (V4.1.0; Supplementary Fig. 2).

### Variants harmonization

Differential call rates resulting from variations in sequencing depth between cases and controls were partially mitigated through the implementation of a previously documented site-based pruning strategy. Briefly, variants were initially filtered if the absolute difference in the proportion of cases relative to controls, both meeting a sufficient call rate threshold for the site, exceeded 0.0178, a threshold derived from the maximum cumulative sum of call rate variances (Supplementary Fig. 3a). Furthermore, we removed 6.0% variants that reached the genome-wide significance threshold (*P* < 5 × 10^−8^) in the call rate association test (two-sided *P* value from Fisher's exact test); Supplementary Fig. 3b).

### Population sub-structure control

Owing to variations in ancestry, geographical regions, and other contributing factors, the genetic composition of the Chinese population is intricate, marked by diverse genetic subgroups and patterns [33, 34]. Our study employed four approaches to account for population substructure within our sample cohort [35]. (1) ADMIXTURE analysis was conducted using European (n = 503, EUR), American (n = 357, AMR), and African (n = 661, AFR) individuals from the 1000 Genome Project (1 KG) [36] as the reference populations. ADMIXTURE analysis was carried out for values of K ranging from 2 to 9 using ADMIXTURE v.1.3 [37]. Among these, K = 4 was identified as the optimal value with the lowest cross-validation error. Individuals with more than 20% probability of assignment to EUR, AMR, or AFR clusters were excluded from the study. (2) We performed iterative random subsampling tests on subset of control individuals to detect outlier SNPs within the population. Each model was subjected to 30 permutations and modeled using linear mixed model (LMM) methods. A total of 4,872,903 SNPs with nominal *P* below 0.05 and exceeding the *P* < 0.05 threshold ten times or more out of the 30 permutations for each model were detected and subsequently removed from the final GWAS LMM summary statistics. (3) Principal-component (PC) analysis with linkage disequilibrium (LD)-independent SNPs (100 kb window, 20 SNPs within each window, at an r^2^ of 0.2) was done with PLINK v1.07 [38]. PC1–PC10 were assessed for their associations with disease phenotype status using a generalized linear model (GLM) and then included in the GWAS as covariates. (4) The genetic relationship/kinship matrix (GRM) was created and integrated into the LMM for final GWAS modeling.

### Genome-wide association analysis

After sample and variants QC, we estimated associations for common variants (MAF > 0.05) with KC using fastGWA and PLINK2, while adjusting for sex, age and the first ten PCs. Genomic control factor (lambda GC) was calculated to evaluate inflation. After association analysis, we identified LD-independent loci using PLINK clumping function (parameters: -clump-p1 = 5 × 10^−8^, -clump-p2 = 0.05, -clump-r2 = 0.4, -clump-kb = 500), and merged the loci with physical overlap using bedtools [39].

### Variants annotation

Gene-based annotations to obtain information about the functional consequences for exonic variants were conducted by Ensembl’s Variant Effect Predictor (VEP v.99) [40] for human genome assembly GRCh38 [41]. Pathogenicity, including pathogenic (P) and likely pathogenic (LP) variants were assigned according to 2015 American College of Medical Genetics (ACMG) criteria using InterVar [42], which is a computational implementation of expert panel recommendations for clinical interpretation of genetic variants (ACMG 2015 criteria) [43]. Variants that were rare (maximum population-specific MAF < 1% in the Genome Aggregation Database) [44], protein-altering (missense, splice site, stop gain, start loss, stoploss) were classified as pathogenic or likely pathogenic. For protein-coding variants, annotation was performed based on four catalogs as outlined in our prior documentation: (1) synonymous; (2) benign missense (B-mis); (3) damaging missense (D-mis); and (4) protein-truncating variants (PTVs). Briefly, using VEP annotations (v.99), missense variants were categorized into “inframe deletion”, “inframe insertion”, “missense variant” or “stop lost” variants. Within the missense variants, one subtype of B-mis variant was predicted as “tolerated” and “benign” by PolyPhen-2 and SIFT, respectively, while another benign mutation displayed a combined annotation dependent depletion (CADD) score < 15. Additionally, D-mis variants were predicted as “probably damaging” and “deleterious” by PolyPhen-2 and SIFT and CADD > 20. Finally, PTVs were classified as “frameshift variant”, “splice acceptor variant”, “splice donor variant”, “stop gained”, or “start lost” variants (Supplementary Information Table 2).

### Excess of rare, deleterious protein-coding variants in individuals with KC

We performed burden tests across the entire exome and biologically relevant gene sets to assess the enrichment of rare variants in individuals with KC, utilizing our previously published pipeline [45]. Briefly, rare genetic variants with a MAF < 0.05 were aggregated into gene-burden tests, employing both Fisher’s exact test and logistic regression. This allowed us to investigate the enrichment of rare variants in individuals with EM as compared to controls. Pre-defined gene sets from the Gene Ontology (GO) biological process ontology, KEGG, REACTOME, and transcription factor targets from The Molecular Signatures Database (MSigDB) [46] were also subjected to evaluation.

### Gene-based collapsing analysis

Our gene-based analysis focused exclusively on deleterious rare variants annotated as PTVs. A total of 392 PTVs, mapped to 250 unique genes, were included in this analysis (Supplementary Information Table 3). To assess whether a particular gene exhibited an enrichment or depletion of rare PTVs in KC cases, we conducted gene-level association tests including Fisher’s exact test, burden analysis and SNP-Set (Sequence) Kernel Association Test (SKAT) [47] with predefined covariates such as principal components (PC1-PC10). The exome-wide correction threshold for multiple testing was established at *P* < 4 × 10^−5^ (0.05/250/5) using Bonferroni correction method. As previously descripted, we generated empirical *P* values by performing 1000 permutations of case–control labels. For each permutation, we ordered the Fisher’s exact test *P* values for all genes and calculated the average across all permutations. This process yielded a rank-ordered estimate of the expected *P* value distribution.

## Results

### Characterization of germline variants in the Chinese KC cohort

**Fig. 1:**
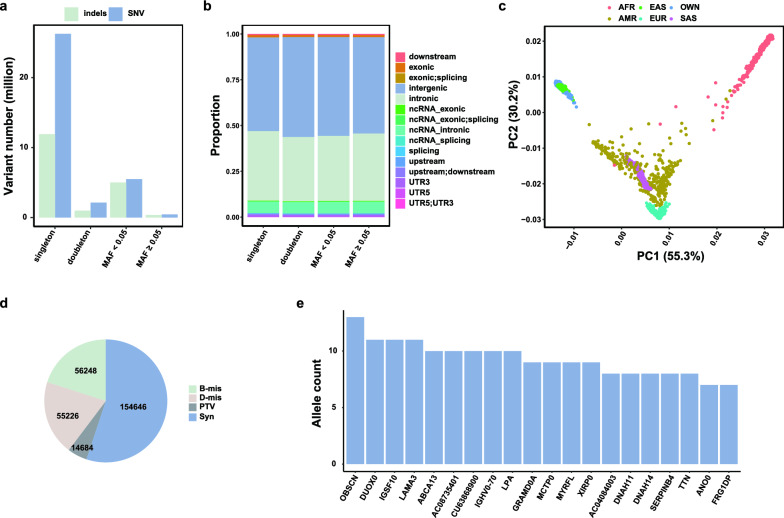
General characterization of germline variants in individuals with keratoconus (KC) and controls. **a** Distribution of indels and single nucleotide variants (SNVs) across the four minor allele frequency (MAF) bins. The bin of ‘‘MAF < 0.05’’ excludes singletons and doubletons. **b** Fraction of variants annotated by RefSeq genomic functions across the four MAF bins. The bin of ‘‘MAF < 0.05’’ excludes singletons and doubletons. **c** Principal Component Analysis plot comparing KC individuals with populations from the 1000 Genomes Project. **d** Fraction of exonic variants annotated by the Variant Effect Predictor (VEP). **e** Distribution of the prevalence of pathogenic alleles among cases. B-mis: benign missense; D-mis: damaging missense; PTVs: protein-truncating variants; Syn: synonymous

From WGS data of 830 individuals with KC and 779 controls, we detected 52,617,611 biallelic variants with mean coverage exceeding 8X, of which 37% were absent from the gnomAD (v4.1.0) database. Remarkably, the majority of these variants (89.8%) were categorized as rare or low-frequency, with a MAF < 0.05 (Fig. 1a). Across all frequency bins, a notable proportion of variants were observed to be annotated as intergenic and intronic (Fig. 1b). After stringent quality control at the sample level, 16 cases and 8 controls were filtered out due to heterozygosity and principal component analysis outliers (Supplementary Fig. 1). The remaining samples were all ancestry-matched, closely resembling East Asian ancestry (Fig. 1c).

Our subsequent focus shifts to variants annotated with coding consequences, which were categorized into four catalogs: 14,684 PTVs, 55,226 D-mis variants, 56,248 B-mis variants, and 154,646 synonymous variants (Fig. 1d). For PTVs, 5893 variants were exclusively detected in the KC groups. These variants were annotated in 4537 genes, among those, *OBSCN* had the highest prevalence of pathogenic alleles in cases (AC = 13, Fig. 1e). The *OBSCN* gene encodes obscurin, a protein involved in the assembly and organization of myofibrils. Notably, the expression level of this gene has been reported to be associated with KC phenotypes and responsive to cyclic mechanical stretch (CMS) [48].

### Analysis of the gene-based burden of rare PTVs for KC

Our WGS data facilitated set-based analyses, allowing for the aggregation of effects from multiple rare variants associated with KC. We restricted the burden test to rare variants covered by sequencing depths of more than 20, yielding a set of 392 high-confidence PTVs spanning 250 genes. As mentioned in our previous study, gene-based analysis was conducted using five methods (Fisher’s exact test, Burden, SKAT, SKAT-O, and SAIGE-GENE) as a robustness check. After adjusting for multiple testing, no gene reached the significance threshold (*P* = 4 × 10^−5^). However, *ZC3H11B* emerged as the top gene prioritized by all methods, and it has been implicated in axial length (AL) and refractive errors [49].

To uncover biological and empirical gene sets enriched for PTVs in KC cases, we conducted collapsing analysis using Fisher’s exact test. This analysis aimed to determine if there is a significant difference between the counts of cases and controls carrying at least one qualifying variant. We identified 43 significant gene sets derived from GO categories and REACTOME. We identified three significant pathways (*P* ≤ 2.2 × 10^−8^) derived from GO categories and REACTOME, after testing them against an empirical distribution generated by repeated sampling of the same number of length-matched genes at random 1000 times (Supplementary Table 1). Our findings suggest that rare PTVs are significantly enriched in biological processes associated with the innate immune response (*P* = 6.84 × 10^−11^) and mRNA metabolic process (*P* = 1.44 × 10^−10^).

### Single-variant association analyses identified novel common susceptibility loci for KC

**Fig. 2:**
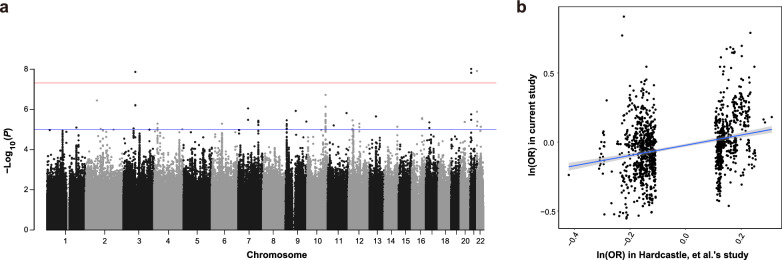
Single-variant association analyses for common variants. **a** Manhattan plot of the linear regression analysis for 814 keratoconus cases and 771 controls. The log_10_ (*P* value) from the final genome-wide association study (GWAS) summary is shown on the y-axis for all single-nucleotide polymorphisms (SNPs) along the different autosomes (x-axis). **b** Comparison of effect size of association for the same SNPs in the largest GWAS meta study and this study

We examined all common variants that passed standard quality control for genome-wide associations in KC, utilizing a LMM-based method (fastGWA) capable of accommodating population structure and relatedness. The association signals were further validated by PLINK, demonstrating high consistency (r = 0.99, *P* < 2.2 × 10^−8^; Supplementary Fig. 4). The genomic inflation factor of 0.99 indicates that the association tests conducted in the GWAS are well-calibrated and not significantly influenced by confounding factors (Supplementary Fig. 5). The discovery analysis identified four variants that reached the genome-wide significance level (*P* ≤ 5 × 10^−8^), including three intergenic and one intronic SNPs near three novel genes (Fig. 2a and Supplementary Table 2). Further studies are needed to determine the function of these three genes in relation to KC.

In addition, our study successfully replicated three previously reported loci at the nominal significance level (*P* ≤ 0.05), namely *EIF3A*, *FNDC3B* and *COL5A1*. Specifically, we identified an upstream variant of *EIF3A*, rs3824830, associated with KC at significance level of *P* = 4.14 × 10^−6^. This SNP demonstrated GWAS-level significance in a meta-analysis study encompassing mixed populations. The direction of the effect size was consistent across both studies. To evaluate the transferability of KC-related signals across populations, we compared effect sizes using suggestive significant SNPs identified in the largest multi-ethnic GWAS of KC. Significant between-population correlation of allelic effects (i.e., logOR) and concordant direction of effect for variants were observed (r = 0.29,* P* < 2.2 × 10^−16^; Fig. 2b).

### Cross-ancestry PRS accounts for a slight yet statistically significant variability between individuals with KC and controls

To determine whether markers identified in the largest KC cohort have predictive value in Chinese individuals, we constructed PRS models using summary statistics from a mixed GWAS meta-analysis. The *P* value thresholding (P + T) method was used to generate 6 predictors according to a set of *P* value selection thresholds as inclusion criteria for SNPs. Then, the “best-fit” PRS was selected using regression to explain the highest phenotypic variance [50]. Specifically, the best model with minimal AIC value was prioritized, demonstrating *r*^2^ = 0.2 and *P* = 1 × 10^−6^, involving 101 SNPs (Supplementary Fig. 6).

**Fig. 3:**
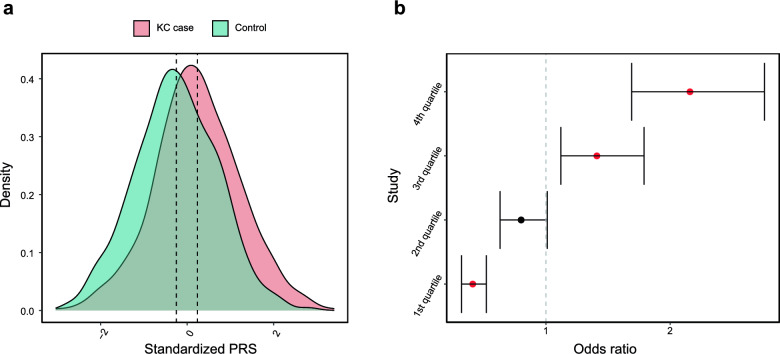
Polygenic risk score for keratoconus (KC) in Chinese cohort. **a** Distribution of polygenic risk score (PRS) between KC cases and controls. The vertical line indicates the mean PRS in each group. **b** Odds ratio (OR) for each quartile (25%) of PRS distribution. Error bar represents the 95% confidence interval of the OR. OR values are relative to the first quartile as baseline

Next, we set out to assess the transferability of PRS and their clinical utility in the Han Chinese population. The distribution of PRS in cases significantly differed from that in controls (t = 10.02, *P* = 2.20 × 10^−16^; Fig. 3a), with individuals in the upper quartile of PRS exhibiting a 2.16 odds ratio (95% CI: 1.69–2.76, *P* = 9.67 × 10^−16^) compared to those in the lowest quartile (Fig. 3b).

## Discussion

Here, we compiled one of the largest cohorts of Han Chinese individuals with KC, genotyped using WGS. This comprehensive dataset enabled a thorough exploration of the genetic landscape and underlying biological mechanisms of KC. Both common and rare variants were included in this analysis to identify ancestry-specific or common signals predisposed to KC. With the benefit of a high-density genotyping panel, previously unknown risk SNPs were identified. Importantly, we observed comparable effect sizes of associations for the same SNPs in individuals of Chinese and multiethnic populations. These findings, along with the transferability of the PRS in stratifying KC risk individuals, indicate high directional concordance of genetic correlations across trans-ancestry samples.

Despite the limited sample size, our study boasts several strengths. First, it employs a genome wide sequencing strategy in the Chinese population, which has been seldom investigated before. WGS provides high-density coverage of non-coding regions, offering an opportunity to identify novel susceptibility loci in specific populations [51]. Since most existing GWAS studies have primarily focused on individuals of European descent [52, 53], understanding the genetic factors underlying KC and capturing broader genetic diversity for clinical translation has been hampered [54]. The unveiling of the largest Chinese KC cohort to date highlights a significant milestone in the field, offering a wealth of insights into the genetic underpinnings and clinical manifestations of this complex condition.

We successfully replicated three KC-associated signals in the Chinese population, namely *COL5A1*, *EIF3A*, and *FNDC3B*. These genes play crucial roles in maintaining the structural integrity and stability of the cornea. Specifically, the *COL5A1* gene encodes one of the key components of type V collagen, which is essential for the structural integrity and elasticity of the cornea [15, 55]. *COL5A1*-related genetic mutations can compromise the structural stability of the corneal matrix, making it more susceptible to mechanical stress. Chronic eye rubbing, a common behavior in individuals with KC, may exacerbate the mechanical strain on the cornea. This mechanical stress can further destabilize the already weakened corneal structure due to *COL5A1* mutations, accelerating the development and progression of KC. *EIF3A* participates in protein translation processes and is implicated in cellular growth and proliferation [56]. *FNDC3B* contributes to cell adhesion and migration, processes crucial for maintaining corneal health. We also discovered two novel synonymous variants, one in *TXNDC2* and the other in *GSTT4*. The low-frequency G allele (MAF = 0.02) of the SNP rs11081510 within the *TXNDC2* gene was first reported to be associated with KC. The SNP exhibited divergent allele frequency distribution between our in-house and public databases. The common SNP, rs7291160, within *GSTT4* was also found to be significant in our study. This gene is implicated in the glutathione metabolic process and is predominantly active in the cytoplasm. Further analysis requires the replication and functional validation of these associations.

Early detection of KC enables clinicians to implement therapeutic measures, such as educating patients to avoid eye rubbing, or in cases of progressive disease timely performing corneal crosslinking. This also allows for informed decisions regarding the suitability of refractive procedures, helping to mitigate the risks associated with ectatic complications from corneal laser surgery. Our study offers the possibility of risk stratification for KC based on genomic data, which is particularly notable for its pioneering observations in the Chinese population. Despite the limited power of our study, we observed a strong concordance in effect size across different ancestries. This stratification could aid in earlier diagnosis and more effective screening for KC. By generating a PRS model from top SNPs using the largest GWAS dataset to date, individuals in the top 25% with identical predispositions exhibited a 2.16-fold higher risk compared to the remainder of the population. This result holds promise but remains unsatisfactory as the risk score relies on allelic effect estimates from other populations, resulting in reduced trans-ethnic performance. Additional studies involving large Chinese cohorts are necessary to jointly model GWAS summary statistics from multiple populations, thereby enhancing cross-population polygenic prediction [57].

We note that although our study mitigates the current Eurocentric biases in KC GWAS, the gap in fully characterizing the genetic architecture and understanding the genetic and nongenetic contributions to KC remains substantial. The limitations of this work primarily include the small sample size for GWAS analysis, which leads to insufficient statistical power and biased effect size estimation. Large-scale studies enable the confident identification of variants with small effect sizes and low allele frequencies, thereby facilitating a deeper understanding of the genetic basis of KC. Another key limitation of our study is the absence of functional validation experiments, both in vitro and in vivo, which are crucial for confirming the biological relevance of the identified genetic variants. Consequently, we are unable to provide direct evidence linking the identified variants to cellular or molecular pathways. This limits our ability to fully interpret the pathophysiological implications of our genetic findings. Next, all newly described genes linked to KC are derived from our in-house dataset. Accordingly, these findings warrant replication in additional cases to further investigate the broader impact of these genes in KC. Finally, our study lacked ophthalmologic testing data for controls, precluding quantitative comparisons of ocular or corneal deficits attributable to disease-related variants.

## Conclusions

We initially conducted a WGS-based GWAS study for KC in a sizable Chinese cohort, which not only enhances the efficiency of array-based genetic studies for the identification of both common and low-frequency susceptibility variants but also helps depict the genetic etiology of KC. These findings identify novel genetic associations for KC that require further replication and underscore the transferability of genetic effects across ancestry. Successful pursuit of subsequent steps will refine current heuristics for clinical decision-making and facilitate early prevention strategies for individuals with KC.

## Supplementary Information

Additional file 1.

Additional file 2.

## Data Availability

Individual-level data are not publicly available due to ethical and legal restrictions related to Wenzhou Medical University. The top 3000 most significant SNPs from the KC GWAS summary statistics can be found in Supplementary Information Table 1. The full dataset reported in this paper is available from the lead author upon reasonable request.

## Abbreviations

KC: Keratoconus
CCT: Central corneal thickness
Kmax: Maximum keratometry
WGS: Whole-genome sequencing
SNPs: Single-nucleotide polymorphisms
HWE: Hardy-Weinberg equilibrium
LD: Linkage disequilibrium
MAF: Minor allele frequency
GQ: Genotype quality
VQSR: Variant quality score recalibration
BWA: Burrows-Wheeler Aligner
VEP: Variant Effect Predictor
ACMG: American College of Medical Genetics
PTVs: Protein-truncating variants
D-mis: Damaging missense
B-mis: Benign missense
LMM: Linear mixed model
PRS: Polygenic risk score
CADD: Combined annotation dependent depletion
GO: Gene Ontology
MSigDB: Molecular Signatures Database
SKAT: SNP-Set (Sequence) Kernel Association Test
