# Supplementary material for: Whole-genome sequencing identifies novel loci for keratoconus and facilitates risk stratification in a Han Chinese population
Source: Eye Vis (Lond). 2025 Jan 6;12:5. doi: 10.1186/s40662-024-00421-1 (PMC11706019; doi:10.1186/s40662-024-00421-1)
Supplement: Supplementary file 1 — Additional file 1. [file 40662_2024_421_MOESM1_ESM.docx]

**Supplementary Table 1.** Top 10 significant pathways identified through burden tests.

| **Gene Set** | **Case**  **Mutated** | **Control**  **Mutated** | **Odds Ratio** | ***P* Adjusted** | ***P* Permutation** |
| --- | --- | --- | --- | --- | --- |
| HMGA1 TARGET GENES | 110 | 24 | 4.863 | 3.071E−11 | 0.528 |
| REACTOME INNATE IMMUNE SYSTEM | 149 | 47 | 3.451 | 6.845E−11 | 0.542 |
| MRNA METABOLIC PROCESS | 114 | 28 | 4.322 | 1.442E−10 | 0.538 |

**Supplementary Table 2.** The most significant single-variant associations for keratoconus identified by genome-wide association study analysis. NA, Not available.

| **CHR** | **POS** | **RS ID** | **BETA** | ***P*** | **Func.refGene** | **InterVar and Evidence** | **Nearest Gene** | **Alt Freq** |
| --- | --- | --- | --- | --- | --- | --- | --- | --- |
| 21 | 10389576 | NA | 1.431 | 9.769E−9 | intergenic | Uncertain significance | LINC01667(dist=568515), BAGE2(dist=23921) | 0.093 |
| 22 | 10778598 | NA | 1.147 | 1.252E−8 | intergenic | Uncertain significance | FRG1FP(dist=161999) | 0.125 |
| 3 | 75706781 | NA | 1.609 | 1.405E−08 | intergenic | Uncertain significance | LINC00960(dist=27478), ZNF717(dist=23180) | 0.076 |
| 21 | 10467321 | NA | 1.236 | 1.538E−08 | intronic | Uncertain significance | BAGE2 | 0.130 |
