# Supplementary material for: Whole-genome sequencing identifies novel loci for keratoconus and facilitates risk stratification in a Han Chinese population
Source: Eye Vis (Lond). 2025 Jan 6;12:5. doi: 10.1186/s40662-024-00421-1 (PMC11706019; doi:10.1186/s40662-024-00421-1)

**Supplementary Figure 1**. Workflow for quality control before GWAS analysis. KC, keratoconus; GWAS, genome-wide association study; HWE, Hardy-Weinberg Equilibrium; SD, standard deviation; MDS, Multidimensional Scaling; PCA, Principal Component Analysis.


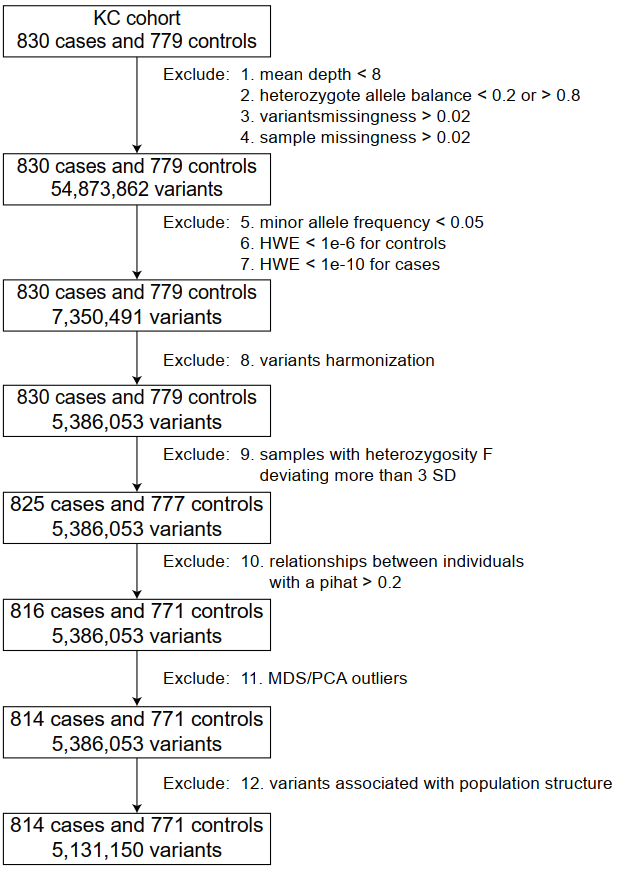


**Supplementary Figure 2**. Workflow for the quality control for rare variants analysis. MAF, minor allele frequency; MAC, minor allele count; gnomAD, Genome Aggregation Database; VEP, Variant Effect Predictor; PTVs, protein-truncating variants; D-mis, damaging missense; B-mis, benign missense; Syn, synonymous; GQ, genotype quality; DP, depth; PTV, protein-truncating variants.


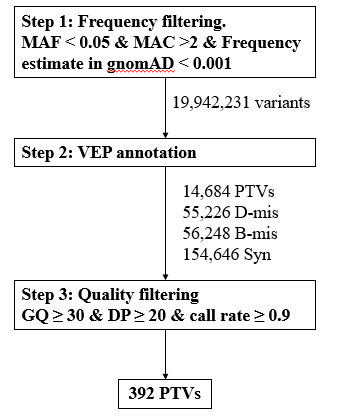


**Supplementary Figure 3**. Quality control for variants harmonization. **a** Cumulative sum of variation plot for site pruning. The y-axis reflects the cumulative sum of variation explained as sites with the largest variation in call rate are pruned (x-axis). The red line represents the point at which we maximize the amount of study-wide variation explained. **b** Manhattan plot shows the association test of call rate between cases and controls by Fisher’s Exact test. Red line: exome-wide significance (*P* < 1×10^−6^).


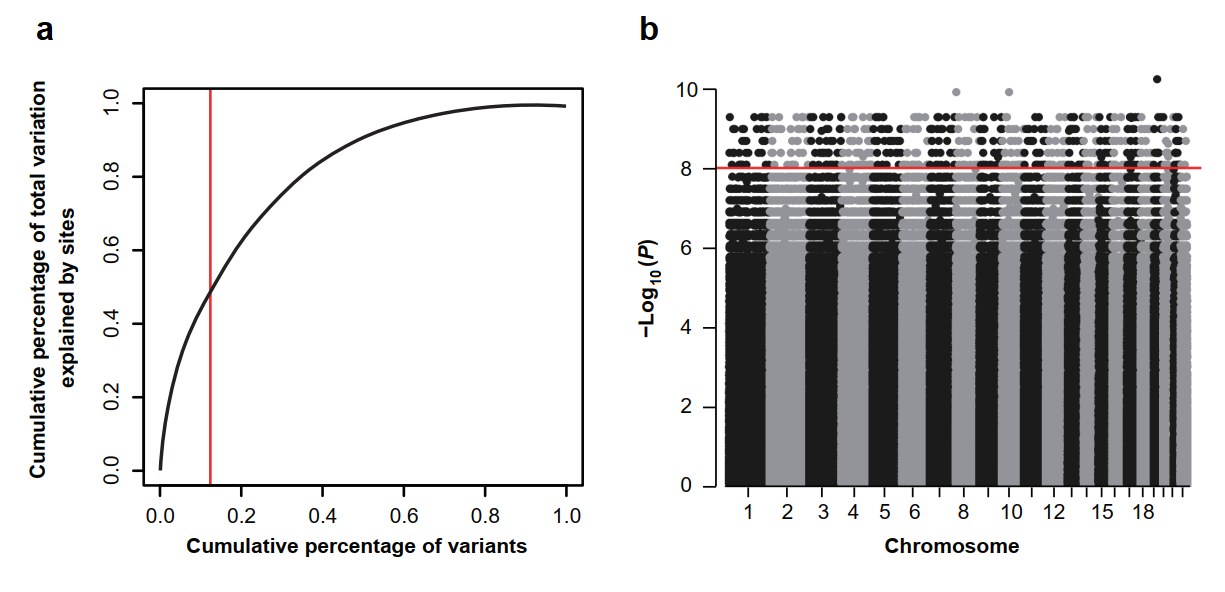


**Supplementary Figure 4**. Pearson correlation between genome-wide association study (GWAS) summary *P* values obtained from two linear regression methods, fastGWA and PLINK.


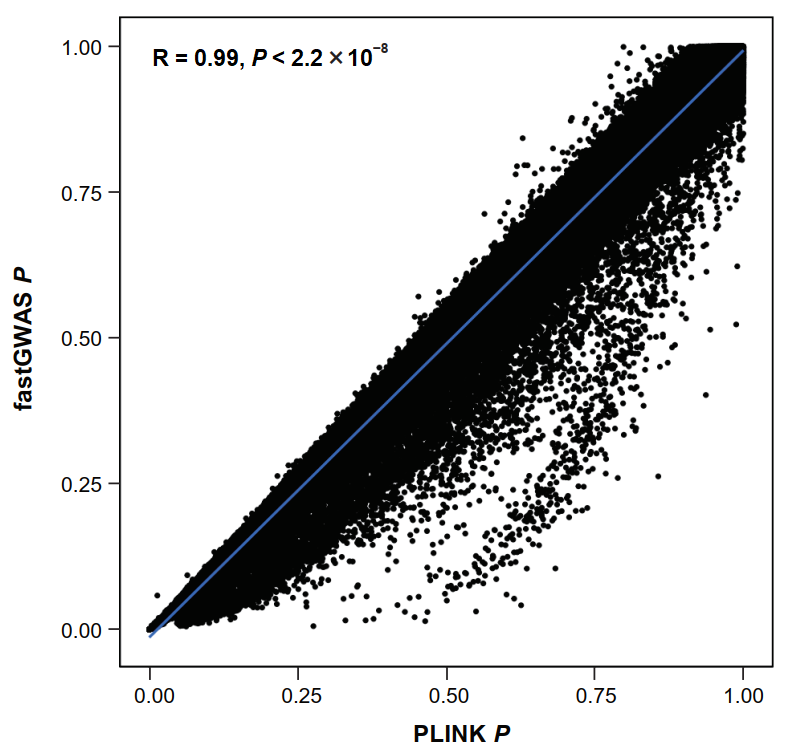


**Supplementary Figure 5**. Quartile-quartile (QQ) plot shows moderate genomic inflation for genome-wide association study.


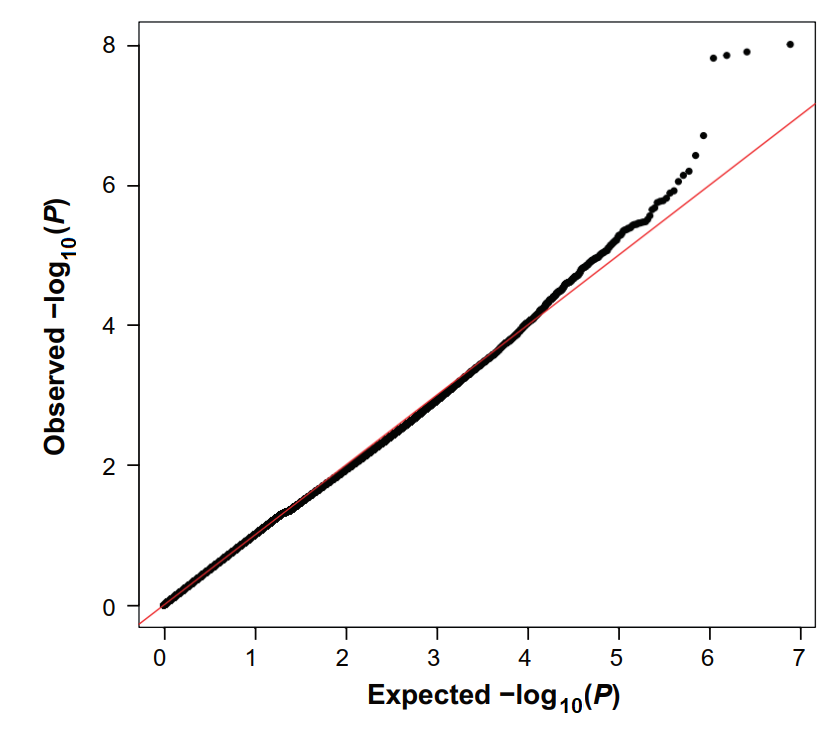


**Supplementary Figure 6**. Evaluation of predictive power of polygenic risk score (PRS) derived from different *P* value threshold on keratoconus. Red stars represent *P* <0.001. AIC, Akaike information criterion.


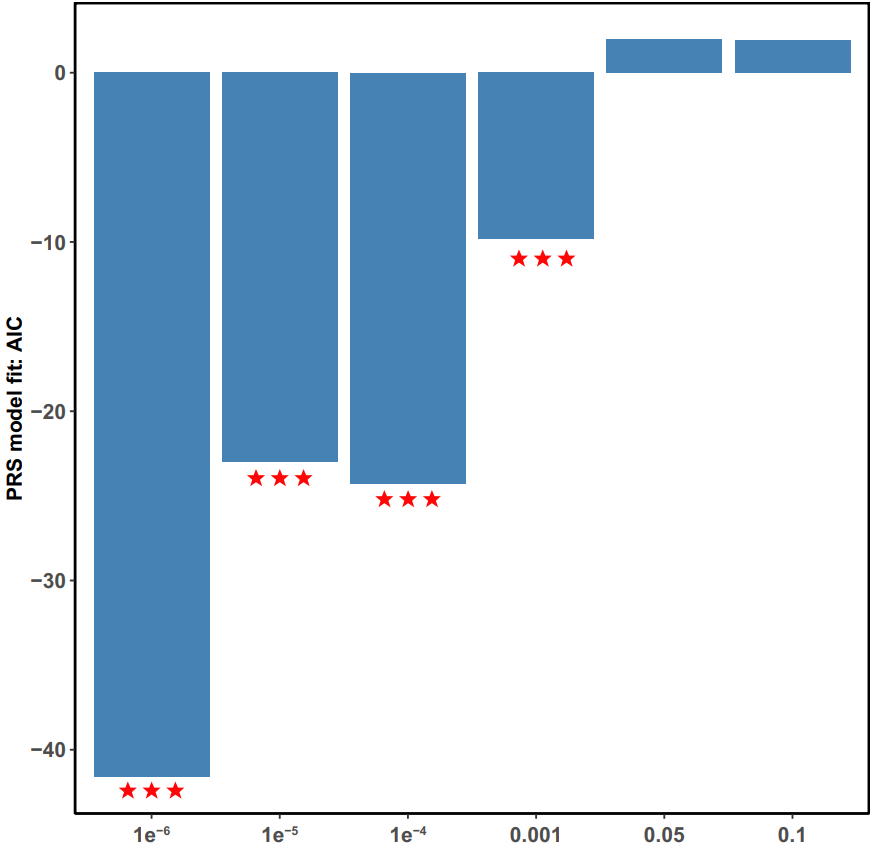

Supplement: Supplementary file 2 — Additional file 2. [file 40662_2024_421_MOESM2_ESM.docx]
